# Clinical conditions and treatment requirements for long‐term survival among hepatitis B‐related hepatocellular carcinoma initially treated with chemoembolization

**DOI:** 10.1002/cam4.2380

**Published:** 2019-07-17

**Authors:** Zhen‐Xin Chen, Zhi‐Wei Jian, Xi‐Wen Wu, Jun‐Cheng Wang, Jing‐Yuan Peng, Xiang‐Ming Lao

**Affiliations:** ^1^ Department of Hepatobiliary and Pancreatic Surgery Sun Yat‐sen University Cancer Center Guangzhou P. R. China; ^2^ State Key Laboratory of Southern China Guangzhou P. R. China; ^3^ Collaborative Innovation Center for Cancer Medicine Guangzhou P. R. China

**Keywords:** clinical characteristics, long‐term survival, transarterial chemoembolization, treatments after chemoembolization

## Abstract

**Objective:**

Transarterial chemoembolization (TACE) is recommended to treat intermediate/advanced stage of hepatocellular carcinoma (HCC). However, the overall survival among initially TACE‐treated patients varies significantly. The clinical characterization of long‐term survival following TACE remains uncertain. We sought to identify clinical parameters and treatment requirements for long‐term survival among patients with hepatitis B‐related HCC who were initially treated with TACE.

**Materials and Methods:**

The included patients with HCC were admitted to our cancer center between December 2009 and May 2015. Patients who survived for >3 years were compared with those who died within 3 years. The clinical and laboratory findings that were associated with the survival were also analyzed.

**Results:**

One in six (17.9%) patients with HCC in this cohort survived for > 3 years after TACE. Body mass index (BMI) ≥ 23kg/m^2^, aspartate aminotransferase levels ≤ 40 U/L, an activated partial thromboplastin time ≤ 34 seconds, α‐fetoprotein (AFP) levels ≤ 25 ng/mL, antiviral therapy, tumor size ≤ 8 cm, solitary nodule, and the absence of vascular invasion were independently favorably associated with a 3‐year survival. An absence of vascular invasion was the only independent factor associated with 3‐year survival in patients who received resection and/or ablation after TACE.

**Conclusion:**

In this cohort, a 3‐year survival was associated with BMI, antivirus treatment, tumor status, hepatic function, and AFP level. Distant metastasis did not negatively impact the long‐term survival among patients with hepatitis B‐related HCC initially treated with TACE. Vascular invasion was the single impediment to long‐term survival in patients who received add‐on resection and/or ablation after TACE.

## 1 INTRODUCTION

Hepatocellular carcinoma (HCC) ranks as the fifth most common cancer and is the second leading cause for all cancer‐related deaths worldwide.^1^ The highest HCC incidence occurs mainly in the Asia‐Pacific region. The HCC burden in China accounts for nearly half of all HCC cases and deaths in the world.^2^, ^3^ Hepatitis B virus (HBV) infection is one of the most important risk factors for HCC and is responsible for approximately 80% of virus‐associated HCC cases in China.^4^ HBV infection contributes to carcinogenesis, cancer recurrence, and poor long‐term survival in HBV‐related HCC.^5^, ^6^, ^7^

In theory, patients with HCC may receive surgical resection, liver transplantation, or tumor ablation as curative therapies. However, these three treatment options all have limitations as follows: only 5%‐10% of patients with HCC are eligible for hepatectomy, as the majority of cases of HCC are diagnosed at the intermediate and advanced stage; Liver transplantation is limited by a severe shortage of donor livers and a high level of perioperative morbidity and mortality; Local tumor ablation is only effective in cases where the tumor size is <5 cm.^8^

Transarterial chemoembolization (TACE) is currently an advised first‐line treatment for patients who have unresectable, large/multifocal HCCs that are not concurrent with vascular invasion or extrahepatic metastasis. This procedure aims to deliver chemotherapeutic agents with mixed lipiodol to the cancer lesions through tumor‐feeding arteries with limited cytotoxic effects on the surrounding liver parenchyma.^2^, ^9^, ^10^ Clinical data suggest that the overall survival (OS) is extended in selected patients with HCC following TACE.^11^, ^12^, ^13^ At early observation, the median survival time (MST) among patients with HCC who are initially treated with TACE was around 20 months.^14^ Currently, improved patient selection methods and optimization of the procedure have extended the median survival to 30‐40 months.^15^, ^16^ However, clinical characterization of long‐term survivors remains uncertain.

In the present study, we assessed the key factors that are associated with a survival period of 3 years among the patients with hepatitis B‐related HCC initially treated with TACE.

## 2 PATIENTS AND METHODS

### 2.1 Patients and inclusion criteria

The clinical data of 1370 patients initially diagnosed with HCC and consecutively received TACE in our cancer center between December 2010 and May 2015 were retrieved and retrospectively assessed. This study protocol was approved by the Institutional Review Board for ethics at our cancer center.

The included patients were stratified into two groups based on the survival time: short‐term (died within 3 years) and long‐term (survived  > 3 years). Short‐ and long‐term survival was also analyzed in a subset of patients who received additional resection and/or ablation after TACE. The results from this subset of patients were compared to the patients who did not receive additional resection and/or ablation.

Baseline laboratory evaluation was performed within 1 week before TACE. These evaluations included serum liver biochemistry (alanine aminotransferase [ALT], aspartate aminotransferase [AST], total bilirubin [TBIL], and albumin [ALB]) tests, α‐fetoprotein (AFP) levels, creatinine levels, prothrombin time (PT), activated partial thromboplastin time (APTT), HBV serology tests of HBsAg, hepatitis B surface antibody, hepatitis B core antibody, hepatitis B e antigen, hepatitis B e antibody, and HBV DNA quantification. The baseline height and body weight of each patient were measured before TACE. The body mass index (BMI) was calculated by dividing the weight (kg) by the height (m) squared, and it is divided into subgroups using the WHO criteria set for the Asian population.^17^ Diagnosis of HCC followed the criteria recommended by the European Association for the Study of the Liver (EASL). Tumor characteristics and Barcelona Clinic Liver Cancer (BCLC) stage were determined using imaging findings and/or the intraoperative observation.

**Figure 1:**
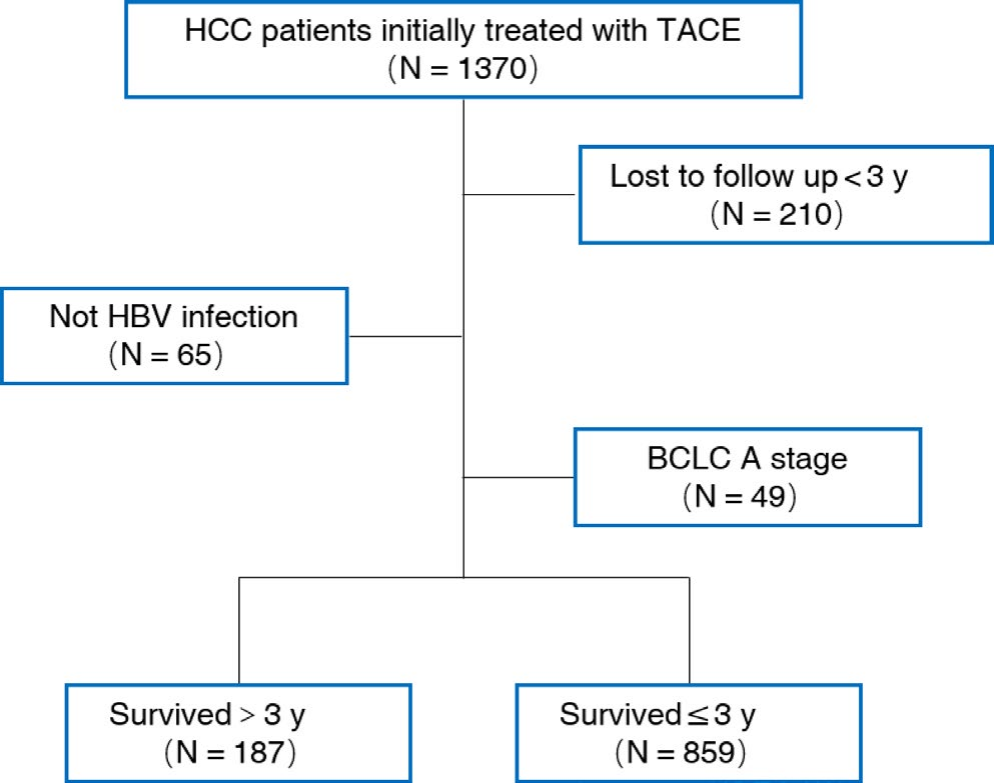
Flowchart of patients enrollment. *BCLC* Barcelona Clinic Liver Cancer staging system; *TACE* transarterial chemoembolization

The inclusion criteria were as follows: patients with HCC were included if they were found to be HBV‐positive (HBV surface antigen [HBsAg]‐positive or detectable HBV DNA), have Child‐Pugh class A or B liver disease, BCLC B or C stage, and were initially treated with TACE. The exclusion criteria were as follows: patients were excluded in the case of other concurrent malignancy or nonmalignant severe illness, Child‐Pugh grade C liver function, any prior HCC treatment and lost to follow‐up within 3 years. A total of 1046 patients were included in the final analysis (Figure 1).

### 2.2 Treatments

#### 2.2.1 TACE procedure

TACE followed the procedure that has been described previously.^18^, ^19^ Briefly, once the catheter tip was advanced to the tumor‐feeding arteries, the radiologist slowly injected one or several chemotherapeutic agents mixed with lipiodol. If the blood flow in the chemoembolized artery net was not blocked, gelatin sponge particles were injected to make sure there was a complete blockage. The selection of anticancer agents and the combinations were individualized for each patient. Our results suggest that the difference in the combinations of anticancer agents that were used was not associated with the long‐term survival (Table S1).

### 2.3 Subsequent treatments

After the initial TACE, the patients were monitored and additional treatments, including repeated TACE, local ablation, hepatectomy, or sorafenib treatment, were performed if they were deemed necessary on a case‐by‐case basis. The additional treatment options were selected based on the tumor burden, liver function, and the patient's preference. Specifically, hepatic resection was performed on patients whose tumor had shrank and a gross residual lesion could potentially be resected. Local ablation (including radiofrequency ablation and microwave ablation) was offered to patients whose residual lesion was ≤3.0 cm in cases where the procedure could potentially eliminate all gross lesions detected radiologically, usually when embolization was technically inaccessible. Repeated TACE at 6‐8 weeks intervals was offered to patients whose residual tumor enhancement and residual tumor vascularity could be seen on CT imaging or hepatic artery angiographs without contraindications to a new round TACE. Contraindications to repeated TACE include: (a) an Eastern Collaborative Oncology Group (ECOG) score >2; (b) deterioration of liver function to Child‐Pugh C; (c) severe extrahepatic disease; (d) portal vein tumor thrombus with complete vessel obstruction; (e) technically inaccessible embolization (exclusive supply of the residual tumors by extrahepatic collateral arteries, the catheter was not able to reach the target hepatic artery, or obstruction of the tumor‐feeding artery); and (f) refusal to participate in subsequent TACE procedures. For patients with tumor progression without contraindications to TACE, a new round TACE combined with sorafenib treatment was recommended. In cases where there was no indication of subsequent treatment requirements, sorafenib application was recommended. Conservative treatments were applied to patients with terminal HCC or an ECOG score >2.^20^ The last follow‐up date was 28 June 2018.

### 2.4 Antivirus treatment

In this study, antivirals (lamivudine, adefovir dipivoxil, telbivudine, entecavir, or interferon) were advised for eligible patients with HCC according to the clinical practice guidelines of chronic hepatitis B by the EASL.^21^ However, patients ultimately made their own decision on antiviral treatment. The serum HBV DNA level of each patient was regularly monitored every 3‐6 months.

### 2.5 Statistical analysis

Demographic data were collected from the included patients. Categorial data were assessed using the Chi‐squared test and Fisher's exact test. Multivariate analysis was performed using logistic regression to identify the possible independent factors associated with the 3‐year survival. OS was calculated using the Kaplan‐Meier method. The Cox proportional hazards model was used for the univariate survival analysis to determine the association between the individual clinical variables and the OS. All variables with *P* < 0.1 after univariate analysis were subsequently subjected to multivariate Cox regression to determine the hazards ratios and the independence of effects. The starting date for OS calculation was the date of TACE treatment and the last date was either the date of death or the date of the last follow‐up. All statistical tests were two‐sided. All statistical tests were performed using SPSS 21.0 (SPSS, Inc, Chicago, IL, USA).

## 3 RESULTS

**Figure 2:**
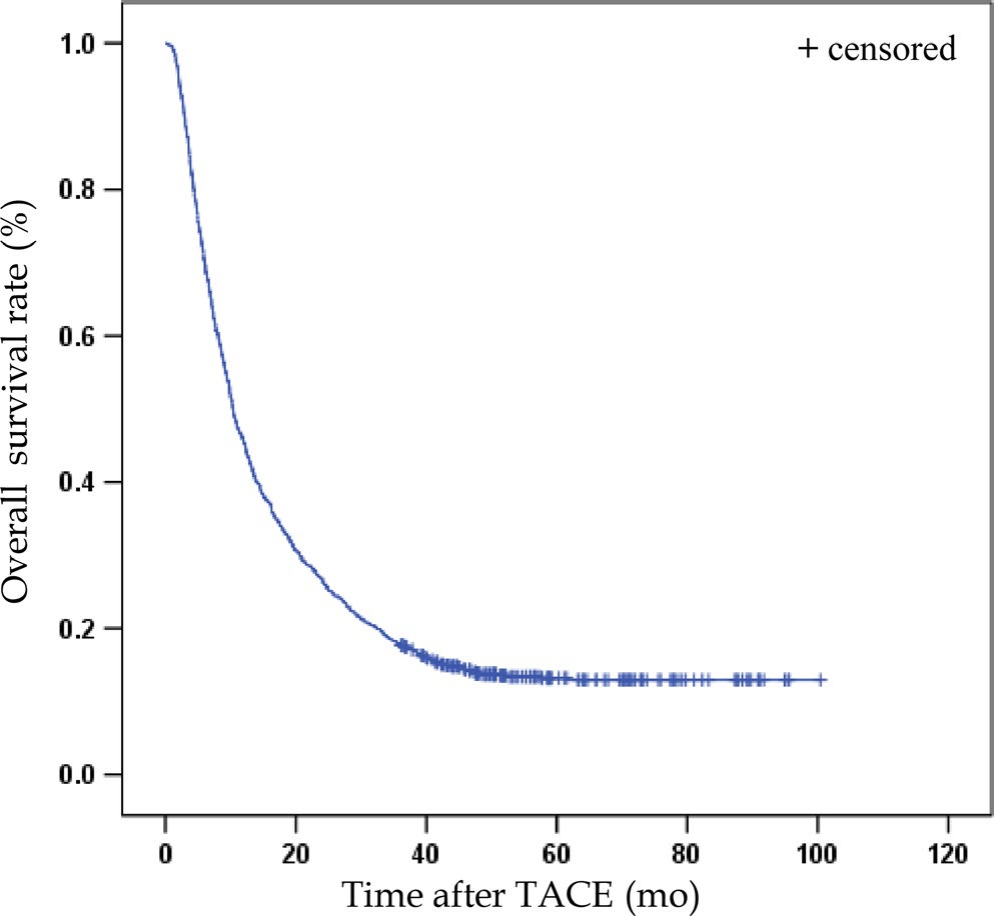
Overall survival curve of 1046 patients with hepatitis B‐related hepatocellular carcinoma (HCC) who were initially treated with chemoembolization

Among the 1370 patients who were initially screened, 1046 of them met the inclusion criteria. The median follow‐up time for those alive was 56.4 months (95% CI, 52.5‐60.3 months). In this cohort, the MST was 10.3 months (95% CI, 9.6‐11.4 months), and the 3‐year survival rate was 17.9% (Figure 2). Significant differences were observed in the BMI, AST, ALB, TBIL, PT, APTT, and AFP levels, the antiviral therapy, tumor size, vascular invasion, metastasis, and BCLC Stages between the short‐ and long‐term survival groups (Table 1).

**Table 1:** Baseline Demographics and Clinical Characteristics of All Hepatitis B‐related HCC Patients Initially Treated with TACE

Characteristic	Short‐term survival≤3 years(n = 859)	Long‐term survival>3 years(n = 187)	*P* value^a^	OR	95% CI	Multivariate analysis *P* value^a^ (logistic regression)
Age (≤45 vs.>45 years)	279:580	51:136	0.165	1.016	0.683‐1.512	0.937
Gender (female: male)	83:776	12:175	0.162	0.620	0.317‐1.213	0.163
BMI (<23 vs. ≥23 kg m^−2^)	549:304	99:88	*0.004*	1.512	1.063‐2.152	*0.022*
ALT (≤40 vs. >40 U/L)	293:566	77:110	0.067	0.992	0.657‐1.496	0.968
AST (≤40 vs. >40 U/L)	164:695	73:114	*<0.001*	0.581	0.372‐0.908	*0.017*
ALB (≤40 vs. >40 U/L)	474:385	79:108	*0.001*	1.313	0.909‐1.896	0.146
TBIL (≤20.5 vs. >20.5 μmol/L)	646:213	159:28	*0.004*	0.700	0.437‐1.121	0.138
PT (≤13.5 vs. >13.5 s)	738:121	171:16	*0.042*	1.133	0.599‐2.144	0.701
APTT (≤34 vs. >34 s)	784:75	183:4	*0.001*	0.231	0.079‐0.678	*0.008*
AFP (≤25 vs. >25 ng/mL)	181:678	75:112	*<0.001*	0.487	0.337‐0.706	*<0.001*
HbsAg (no: yes)	102:746	23:159	0.819			
HBV DNA (≤10000 vs. >10000)	379:480	88:99	0.464			
Antivirus (no: yes)	586:273	97:90	*<0.001*	2.058	1.443‐2.933	*<0.001*
Antivirus agents			0.485			
Lamivudine	44	18				
Adefovir	7	5				
Entecacir	172	48				
Telbivudine	44	16				
Interferon	1	0				
Lamivudine+ Adefovir	4	3				
Adefovir+ Entecacir	1	0				
Tumor size (<8: ≥8 cm)	313:546	108:79	*<0.001*	0.490	0.334‐0.720	*<0.001*
Tumor quantity (solitary: multiple)	352:507	91:96	0.054	0.511	0.352‐0.742	*<0.001*
Vascular invasion (no: yes)	523:336	162:25	*<0.001*	0.278	0.173‐0.446	*<0.001*
Metastasis (no: yes)	790:69	182:5	*0.010*	0.457	0.172‐1.212	0.115
BCLC_Stage (B:C)	489:370	160:27	*<0.001*			
Child_Pugh_Score (A: B)	836:23	186:1	0.102			

Abbreviations: AFP, α‐fetoprotein; ALB, serum albumin; ALT, alanine aminotransferase; APTT, activated partial thromboplastin time; AST, aspartate aminotransferase; BCLC, Barcelona Clinic Liver Cancer; BMI, body mass index; CI, confidence interval; HBV, hepatitis B virus; HbsAg, HBV surface antigen; HCC, hepatocellular carcinoma; HCV, hepatitis C virus; OR, odds ratio; PT, prothrombin time; TACE, transcatheter arterial chemoembolization; TBIL, total bilirubin.

a The italic values indicated statistical significance.

**Figure 3:**
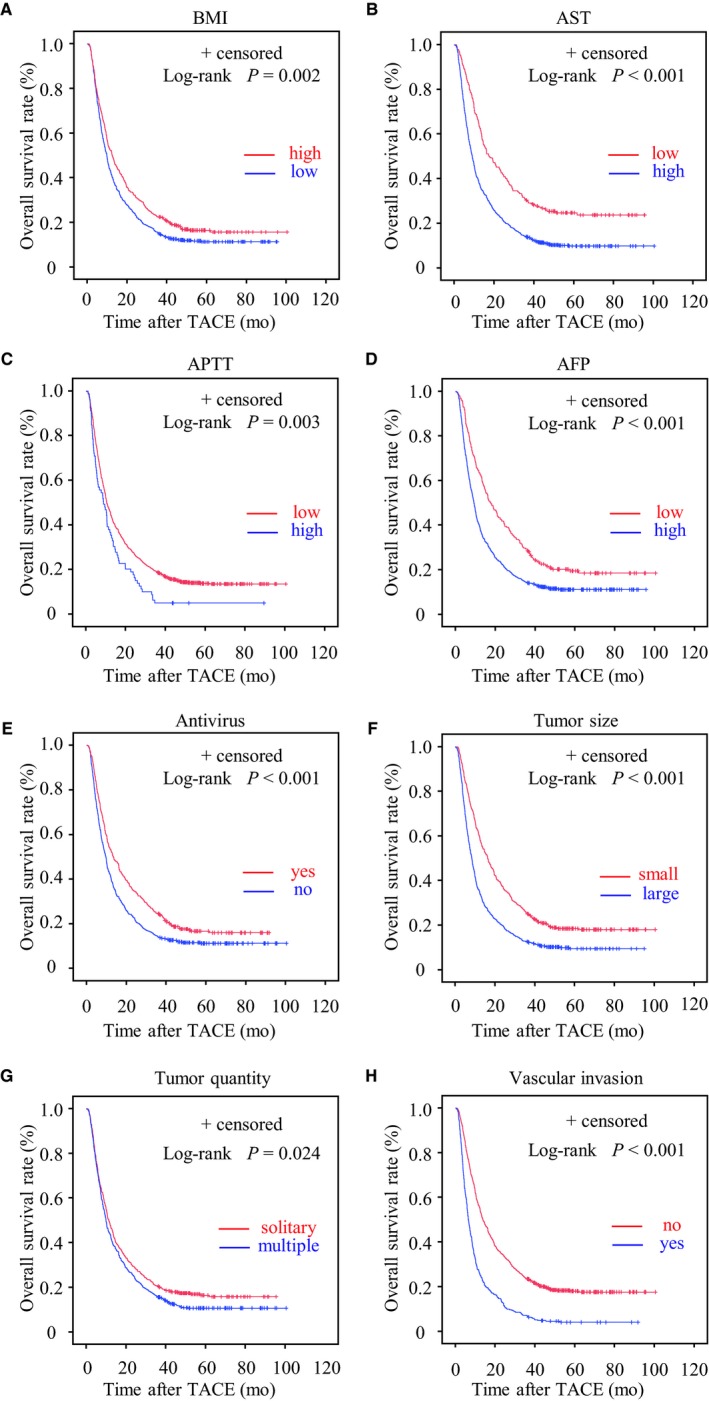
Kaplan‐Meier curves of the overall survival (OS) for 1046 patients with hepatocellular carcinoma (HCC) according to different risky factors: (A) body mass index (BMI, <23 vs ≥23 kg/m^2^), higher BMI was associated with longer OS (*P* = 0.002); (B) aspartate aminotransferase (AST, ≤40 vs >40 U/L), lower AST was associated with longer OS (*P* < 0.001); (C) activated partial thromboplastin time (APTT, ≤34 vs >34 s), shorter APTT was associated with longer OS (*P* = 0.003); (D) α‐fetoprotein (AFP, ≤25 vs >25 ng/mL), lower AFP was associated with longer OS (*P* < 0.001); (E) antivirus treatment (no vs yes), antivirus treatment was associated with longer OS (*P* < 0.001); (F) tumor size (≤8 vs >8 cm), smaller tumor size was associated with longer OS (*P* < 0.001); (G) tumor quantity (solitary vs. multiple), solitary tumor was associated with longer OS (*P* = 0.024); (H) vascular invasion (no vs yes), the absence of vascular invasion was associated with longer OS (*P* < 0.001)

Multivariate analysis (logistic regression model), as shown in Table 1, revealed that there were several independent factors associated with the 3‐year survival, including a higher BMI (OR 1.512, *P* = 0.022), lower AST (OR 1.720, *P* = 0.017), shorter APTT (OR 4.327, *P* = 0.008), lower AFP (OR 2.052, *P* < 0.001), antivirus treatment (OR 2.058, *P* < 0.001), smaller tumor size (OR 2.041, *P* < 0.001), a solitary tumor (OR 1.958, *P* < 0.001), and the absence of vascular invasion (OR 3.602, *P* < 0.001). Kaplan‐Meier analysis and univariate and multivariate analyses (Cox's proportional hazards model) were performed to verify the association between these factors and the OS. Kaplan‐Meier analysis demonstrated that the aforementioned factors were relevant to the OS in HBV‐related HCC patients (Figure 3). Univariate and multivariate analyses (Cox's proportional hazards model) revealed that these factors independently contributed to the prognosis of HBV‐related HCC patients (Table 2). However, distant metastasis did not negatively impact the long‐term survival.

**Table 2:** Univariate and multivariate analysis of factors related to survival using Cox proportional hazards model in all Hepatitis B‐related HCC patients initially treated with TACE

Variable	Univariate analysis*P* value^a^	Multivariate analysis		
		Hazard ratio	95% CI	*P* value
BMI (<23 vs. ≥23 kg m^−2^)	*0.002*	0.869	0.799‐1.080	*0.047*
AST (≤40 vs. >40 U/L)	*<0.001*	1.419	1.108‐1.633	*<0.001*
APTT (≤34 vs. >34 s)	*0.003*	1.337	0.852‐1.429	*0.017*
AFP (≤25 vs. >25 ng/mL)	*<0.001*	1.421	1.150‐1.594	*<0.001*
Antivirus (no vs. > yes)	*<0.001*	0.719	1.006‐1.413	*<0.001*
Tumor size (≤8 vs. >8 cm)	*<0.001*	1.493	1.150‐1.594	*<0.001*
Tumor quantity (solitary: multiple)	*0.024*	1.373	1.595‐2.139	*<0.001*
Vascular invasion (no vs. yes)	*<0.001*	1.843	0.673‐0.895	*<0.001*

Abbreviations: AFP, α‐fetoprotein; APTT, activated partial thromboplastin time; AST, aspartate aminotransferase; BMI, body mass index; CI, confidence interval; TACE, transcatheter arterial chemoembolization.

a The italic values indicated statistical significance.

**Figure 4:**
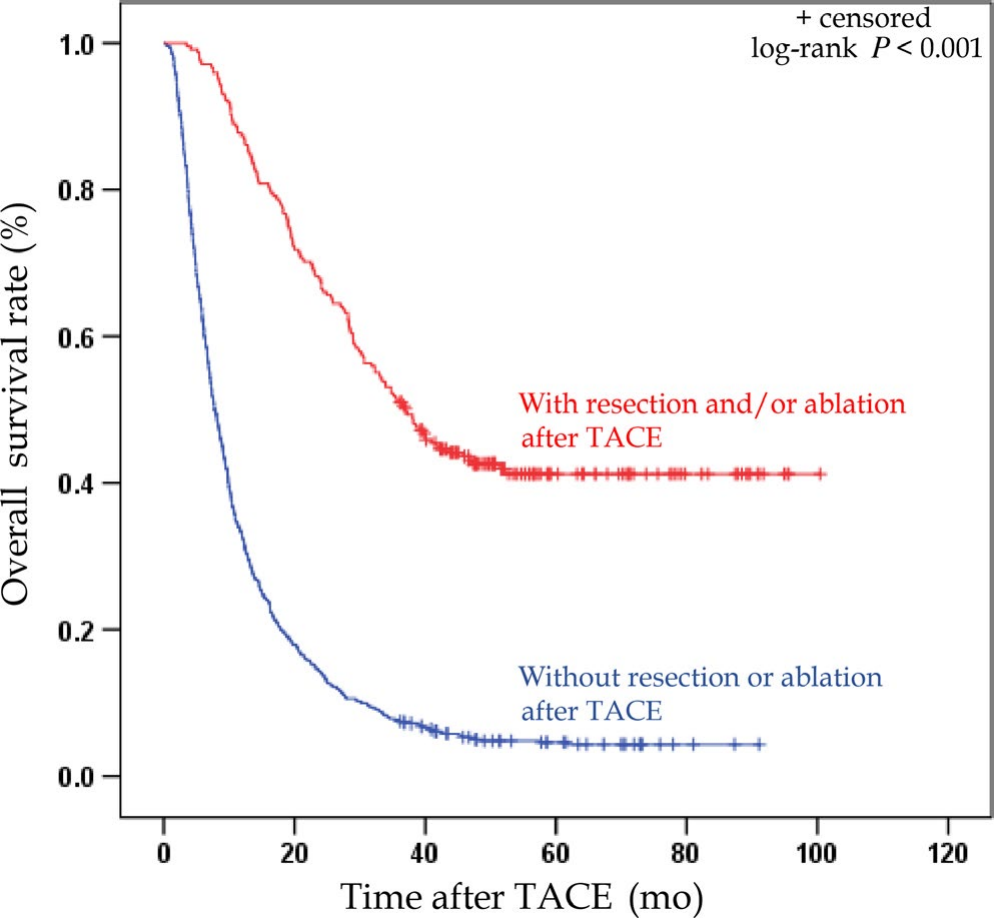
Kaplan‐Meier curves of the overall survival (OS) for 1046 patients with hepatocellular carcinoma (HCC) with or without resection and/or ablation after transarterial chemoembolization (TACE). HCC patients with resection and/or ablation after TACE have longer OS (*P* < 0.001) than HCC patients without resection and/or ablation after TACE

The percentage of patients who reached a 3‐year survival time was significantly higher in the group that received add‐on treatment of resection and/or ablation after TACE than in the patients who did not (Table 3). The survival time was longer in the patients with additional resection and/or ablation (n = 245) after TACE than those who did not (n = 801). The MST of the patients without additional resection and/or ablation after TACE was 7.8 months (95% CI, 7.1‐8.5 months; Figure 4), and only 7.6% of these 801 patients who did not receive additional resection and/or ablation reached a survival period of 3 years. Independent factors that were associated with a 3‐year survival time as shown by multivariate analysis (logistic regression model) (Table 4) included a lower AST (OR 1.944, *P* = 0.033), lower AFP (OR 3.404, *P* < 0.001), smaller tumor size (OR 2.417, *P* = 0.005), solitary tumor (OR 2.131, *P* = 0.014), and the absence of vascular invasion (OR 2.271, *P* = 0.021).

**Table 3:** Differences in Survival rates between Patients with and without add‐on Treatments after Initial TACE

Characteristic	Short‐term survival≤3 years(n = 859)	Long‐term survival>3 years(n = 187)	*P* value
Resection after TACE (no: yes)	798:61	101:86	*<0.001*
Ablation after TACE (no: yes)	792:67	128:59	*<0.001*
Resection and/or ablation after TACE (no: yes)	740:119	61:126	*<0.001*

Abbreviation: TACE, transcatheter arterial chemoembolization.

The italic values indicated statistical significance.

**Table 4:** Identification of Demographic and Clinical Factors Associated with Long‐term Survival among patients without resection and/or ablation after TACE

Characteristic	Short‐term survival≤3 years(n = 740)	Long‐term survival>3 years(n = 61)	*P* value^a^	OR	95% CI	Multivariate analysis *P* value^a^ (logistic regression)
Age (≤45 vs.>45 years)	240:500	10:51	*0.009*	2.036	0.966‐4.293	0.062
Gender (female: male)	71:669	2:59	0.099	0.277	0.063‐1.211	0.088
BMI (<23 vs. ≥23 kg m^−2^)	486:248	36:25	0.255			
ALT (≤40 vs. >40 U/L)	248:492	26:35	0.149			
AST (≤40 vs. >40 U/L)	126:614	26:35	*<0.001*	0.515	0.279‐0.949	*0.033*
ALB (≤40 vs. >40 U/L)	421:319	25:35	*0.031*	1.462	0.810‐2.641	0.208
TBIL (≤20.5 vs. >20.5 μmol/L)	546:194	52:9	*0.048*	0.591	0.271‐1.286	0.185
PT (≤13.5 vs. >13.5 s)	634:106	56:5	0.183			
APTT (≤34 vs. >34 s)	675:65	60:1	0.051	0.159	0.021‐1.216	0.076
AFP (≤25 vs. >25 ng/mL)	155:585	33:28	*<0.001*	0.294	0.166‐0.519	*<0.001*
Antivirus (no: yes)	530:210	44:17	0.932			
Tumor size (<8: ≥8 cm)	245:495	35:26	*<0.001*	0.414	0.223‐0.767	*0.005*
Tumor quantity (solitary: multiple)	295:445	31:30	0.094	0.469	0.256‐0.859	*0.014*
Vascular invasion (no: yes)	436:304	49:12	*0.001*	0.440	0.220‐0.883	*0.021*
Metastasis (no: yes)	674:66	57:4	0.644	0.999	0.325‐3.068	0.998
Child_Pugh_Score (A: B)	719:21	61:0	0.396			

Abbreviations: AFP, α‐fetoprotein; ALB, serum albumin; ALT, alanine aminotransferase; APTT, activated partial thromboplastin time; AST, aspartate aminotransferase; BMI, body mass index; OR, odds ratio; PT, prothrombin time; TACE, transcatheter arterial chemoembolization; TBIL, total bilirubin.

a The italic values indicated statistical significance.

Among the 245 patients with HCC who received add‐on resection and/or ablation after TACE, the MST was 37.1 months (95% CI, 31.1‐43.1 months) and the 3‐year survival rate reached 51.4% (Figure 4). The multivariate analysis (logistic regression model) indicated that the absence of vascular invasion was the only factor that was associated with a 3‐year survival time (Table 5).

**Table 5:** Identification of Factors Associated with Long‐term Survival among Patients with Add‐on Resection and/or Ablation after TACE

Characteristic	Short‐term survival≤3 years(n = 119)	Long‐term survival>3 years(n = 126)	*P* value^a^	OR	95% CI	Multivariate analysis *P* value^a^ (logistic regression)
Age (≤45 vs.>45 years)	39:80	41:85	0.969	0.853	0.474‐1.534	0.596
Gender (female: male)	12:107	10:116	0.557	0.776	0.308‐1.957	0.591
BMI (<23 vs. ≥23 kg m^−2^)	63:56	63:63	0.645			
ALT (≤40 vs. >40 U/L)	45:74	51:75	0.670			
AST (≤40 vs. >40 U/L)	38:81	47:79	0.378			
ALB (≤40 vs. >40 U/L)	53:66	53:73	0.696			
TBIL (≤20.5 vs. >20.5 μmol/L)	100:19	107:19	0.848			
PT (≤13.5 vs. >13.5 s)	104:15	115:11	0.325			
APTT (≤34 vs. >34 s)	109:10	123:3	*0.046*	0.335	0.086‐1.308	0.116
AFP (≤25 vs. >25 ng/mL)	26:93	42:84	*0.045*	0.607	0.334‐1.101	0.100
Antivirus (no: yes)	53:63	53:73	0.432			
Tumor size (≤8:>8 cm)	68:51	73:53	0.900	1.053	0.590‐1.879	0.862
Tumor quantity (solitary: multiple)	57:62	60:66	0.965	0.903	0.510‐1.599	0.726
Vascular invasion (no: yes)	87:32	113:13	*0.001*	0.334	0.159‐0.703	*0.004*
Metastasis (no: yes)	116:3	125:1	0.358	0.536	0.050‐5.738	0.607
Child_Pugh_Score (A: B)	117:2	125:1	0.613			

Abbreviations: AFP, α‐fetoprotein; ALB, serum albumin; ALT, alanine aminotransferase; APTT, activated partial thromboplastin time; AST, aspartate aminotransferase; BMI, body mass index; OR, odds ratio; PT, prothrombin time; TACE, transcatheter arterial chemoembolization; TBIL, total bilirubin.

a The italic values indicated statistical significance.

## 4 DISCUSSION

Our analysis shows that the achievement of 3‐year survival time in patients with hepatitis B‐related HCC initially treated with TACE was associated with a higher BMI, lower AST, shorter APTT, lower AFP, antivirus treatment, smaller tumor size, solitary tumor, and the absence of vascular invasion. Surprisingly, distant metastasis did not negatively impact the 3‐year survival in this cohort. The absence of vascular invasion was the only factor that was associated with long‐term survival among the patients with add‐on resection and/or ablation after TACE.

A previous study characterized the factors that were associated with long‐term survival among patients with HCC who underwent partial hepatectomy.^22^ In fact, radical resection can only be applied to a small portion of patients with HCC, while TACE can be performed in a larger proportion of patients with HCC. In previous studies concerning the prognosis of HCC treated with resection, “10 years” is commonly considered as the appropriate cutoff value indicating the long‐term survival.^23^, ^24^, ^25^, ^26^ However, as to unresectable HCC patients initially treated with TACE, there is no definite consensus on the appropriate cutoff value to define their “long‐term survival.” As a reference, the 10‐year survival rate of patients undergoing hepatectomy ranges from 15% to 20%,^23^, ^24^, ^25^, ^26^ comparable to 17.9%—the 3‐year survival rate of patients with unresectable HCC in our current study. In addition, the cutoff value is not recommended to dispose beyond the outer 10% of the continuous covariate distribution, namely, years survival rate below 10% in this study, avoiding small numbers in one of the groups following dichotomization, and the substantial losses in statistical power.^27^, ^28^ In the current study, the 4‐ and 5‐year survival rates of patients were 9.9% and 5.4%, respectively, neither statistically appropriate for the cutoff value. Moreover, although not explicitly stated, a 3‐year survival time is usually defaulted to be an important watershed for the prognosis of unresectable HCC patients treated with TACE. And, many previous studies utilized “3 year” as an important time point to report the accordingly survival rate in HCC patients treated with TACE.^29^, ^30^, ^31^, ^32^, ^33^, ^34^, ^35^ Therefore, based on the previous studies and data in the current study, we considered “3 years” as a reasonable (clinically, statistically, and empirically) cutoff value indicating the long‐term survival in HCC patients undergoing TACE. To our best knowledge, our current study represents the first study that identifies the clinical characteristics associated with long‐term survival (using 3‐year survival as a cutoff value) in patients with unresectable HBV‐related HCC (HCC of BCLC stage B or C) treated with TACE.

Our results suggest that a higher BMI may be a favorable factor for long‐term survival. Obesity, with metabolic syndrome, may trigger the development of hepatic steatosis, fibrosis, or cirrhosis leading to HCC.^36^, ^37^, ^38^ However, the relationship between BMI and the prognosis of patients with HCC remains controversial. Some studies including our current study suggested that overweight is associated with a long OS in patients with HCC,^39^ while other studies reported that overweight had either no effect or even a negative effect on patients’ OS.^40^, ^41^, ^42^, ^43^ Recently, Tachi et al demonstrated that lower BMI was associated with severe skeletal muscle volume loss and skeletal muscle fat deposition in patients with chronic liver disease who developed HCC.^44^, ^45^ It may explain why lower BMI was identified as an independent factor impeding long‐term survival of patients with HBV‐related HCC in our current study. Understandably, patients with HCC who have a low BMI may not have sufficient nutritional and physiologic reserve to afford huge energy consumption that results from the overgrowth of cancer cells,^46^, ^47^ or they may have experienced more frequent treatment interruptions due to health deterioration,^48^ leading to a relatively short survival time.

In this cohort, antiviral treatment appeared to aid the achievement of a long‐term survival. Previous studies have indicated that antiviral treatment increases the disease‐free survival and OS in patients with HCC treated with TACE or resection.^20^, ^49^, ^50^, ^51^ A high serum HBV DNA level has been identified as a risk factor for poor prognosis.^52^ In this study, antiviral treatment appeared to suppress HBV replication, mitigate liver injury, and slow down the progression of liver disease (Table.S2), supporting the findings of previous studies.^53^, ^54^ Thus, antiviral treatment relieves the HBV infection/replication‐imposed burden on the HCC lesioned liver and helps to achieve long‐term survival.

However, distant metastasis, a major component in malignant tumor (TNM) staging system, was not independently associated with the short‐term survival in this cohort. Statistically, over 60% of patients with HCC died of liver failure, caused by the progressive intrahepatic lesions, as opposed to 20% of Stage IV patients with HCC who died from respiratory failure caused by metastatic lesions.^55^ These findings might explain why distant metastasis may function as a conditional factor that could negatively impact the long‐term survival in patients with HCC. An intensified treatment of intrahepatic lesions could be more critical for Stage IV HCC. Thus, we cautiously suggest that distant metastasis might not be an absolute contraindication to TACE.

Consistent with previously studies, our analysis demonstrates that an add‐on resection or ablation after initial TACE significantly extended the survival time and increased the percentage of patients who reached a 3‐year survival time.^56^, ^57^, ^58^, ^59^ This suggests that add‐on resection/ablation works synergistically with TACE. TACE reduces or stabilizes the size of large HCCs and induces ischemia and inflammatory edema in tumor tissues, which provide favorable conditions for the success of add‐on resection or ablation treatment. In addition, the add‐on resection or ablation removes or necrotizes hypovascular HCC lesions that are refractory to cytotoxicity by TACE delivered chemicals.^57^, ^58^

In further analysis, vascular invasion was the only risk factor that compromised the long‐term survival in patients who received the add‐on resection and/or ablation after TACE. This finding reveals that the efficacy of the add‐on resection and/or ablation is effective in eliminating almost all of the factors that are required to achieve the 3‐year survival in the group without resection and/or ablation after TACE. The add‐on resection and/or ablation significantly reduced the uncertainty of the HCC outcome and was only impacted by the vascular invasion. We strongly recommend the add‐on resection and/or ablation after TACE whenever the patient is eligible.^48^, ^50^, ^60^, ^61^

A limitation of this retrospective study is that the patients were all recruited from a single center. However, our results are encouraging and will be helpful in future studies designed to verify or extend our findings to improve the prognosis of unresectable HCC treated with TACE.

In summary, our findings suggest that patients with HCC who have higher BMI, normal liver function, lower AFP level, the absence of vascular invasion, smaller tumor size, and solitary tumors may have a better outcome after TACE. In addition, antiviral treatment should be recommended to HBV‐related HCC patients as this may contribute to the achievement of 3‐year survival. However, these factors, excluding vascular invasion, may no longer play a role in the survival time if an add‐on resection or ablation is performed after TACE. Our findings strongly favor an add‐on resection or ablation in cases where the patient is deemed eligible.
