# Supplementary material for: Clinical conditions and treatment requirements for long‐term survival among hepatitis B‐related hepatocellular carcinoma initially treated with chemoembolization
Source: Cancer Med. 2019 Jul 17;8(11):5097–107. doi: 10.1002/cam4.2380 (PMC6718579; doi:10.1002/cam4.2380)
Supplement: Supplementary file 1 [file CAM4-8-5097-s001.docx]

**Table S1. Anti-Cancer Therapeutics used in the treatment of TACE among All Enrolled Patients**

| **Chemotherapy agents** | **Short-term survival**  **≤3 years**  **(n = 859)** | **Long-term survival**  **>3 years (n = 187)** | ***P*-value** |
| --- | --- | --- | --- |
|  |  |  |  |
| **Pharmorubicin RD** | 70 | 20 | 0.707 |
| **Lobaplatin** | 1 | 0 |  |
| **5-Fluorouracil** | 4 | 1 |  |
| **Oxaliplatin** | 0 | 1 |  |
| **Pharmorubicin RD+ Mitomycin** | 9 | 2 |  |
| **Pharmorubicin RD+ Lobaplatin** | 16 | 2 |  |
| **Pharmorubicin RD+5-Fluorouracil** | 3 | 2 |  |
| **Pharmorubicin RD+ Carboplatin** | 10 | 3 |  |
| **Pharmorubicin RD+ Pirarubicin** | 1 | 0 |  |
| **Mitomycin+ Lobaplatin** | 11 | 3 |  |
| **Mitomycin+5-Fluorouracil** | 1 | 0 |  |
| **Mitomycin+ Carboplatin** | 4 | 0 |  |
| **Mitomycin+ Doxorubicin** | 1 | 0 |  |
| **Mitomycin+ Cisplatin** | 2 | 0 |  |
| **Lobaplatin+5-Fluorouracil** | 2 | 0 |  |
| **5-Fluorouracil+ Carboplatin** | 1 | 1 |  |
| **Pharmorubicin RD+ Mitomycin+ Lobaplatin** | 545 | 122 |  |
| **Pharmorubicin RD+ Mitomycin+ 5-Fluorouracil** | 6 | 1 |  |
| **Pharmorubicin RD+ Mitomycin+ Carboplatin** | 118 | 25 |  |
| **Pharmorubicin RD+ Lobaplatin+5-Fluorouracil** | 1 | 0 |  |
| **Pharmorubicin RD+ Lobaplatin+ Pirarubicin** | 1 | 1 |  |
| **Pharmorubicin RD+ 5-Fluorouracil+ Carboplatin** | 46 | 2 |  |
| **Mitomycin+ Lobaplatin+5-Fluorouracil** | 1 | 0 |  |
| **Mitomycin+ Lobaplatin+ Adriamycin** | 2 | 1 |  |
| **Mitomycin+ Carboplatin + Pirarubicin** | 1 | 0 |  |
| **Pharmorubicin RD+ Mitomycin+ 5-Fluorouracil+ Carboplatin** | 1 | 0 |  |
| **Pharmorubicin RD+ Mitomycin+ Carboplatin+ Doxorubicin** | 1 | 0 |  |

**Table S2. Changes in Serum HBV DNA Level between Baseline and Follow-up in both Antiviral Treated and Untreated Groups**

|  | **Antiviral treatment (n = 164)** | | |  | **Untreated (n = 254)** | | |
| --- | --- | --- | --- | --- | --- | --- | --- |
|  | Baseline | Follow-up | *P* value |  | Baseline | Follow-up | *P* value |
| HBV DNA (IU/ml) | 7.9×10^6^ | 1.8×10^5^ | <0.001 |  | 1.5×10^6^ | 9.1×10^5^ | 0.277 |

HBV, hepatitis B virus.
